# Measurement and monitoring patient safety in prehospital care: a systematic review

**DOI:** 10.1093/intqhc/mzab013

**Published:** 2021-01-18

**Authors:** Paul O’connor, Roisin O’malley, Anne-Marie Oglesby, Kathryn Lambe, Sinéad Lydon

**Affiliations:** Discipline of General Practice, School of Medicine, National University of Ireland Galway, Galway H91 TK33, County Galway, Ireland; Irish Centre for Applied Patient Safety and Simulation, National University of Ireland Galway, Galway H91 TK33, County Galway, Ireland; Discipline of General Practice, School of Medicine, National University of Ireland Galway, Galway H91 TK33, County Galway, Ireland; Irish Centre for Applied Patient Safety and Simulation, National University of Ireland Galway, Galway H91 TK33, County Galway, Ireland; Health Protection and Surveillance Centre, 25-27 Middle Gardiner St, Dublin 1, Ireland; Discipline of General Practice, School of Medicine, National University of Ireland Galway, Galway H91 TK33, County Galway, Ireland; Irish Centre for Applied Patient Safety and Simulation, National University of Ireland Galway, Galway H91 TK33, County Galway, Ireland; Irish Centre for Applied Patient Safety and Simulation, National University of Ireland Galway, Galway H91 TK33, County Galway, Ireland; School of Medicine, National University of Ireland Galway, Galway H91 TK33, County Galway, Ireland

**Keywords:** emergency medical services, systematic review, safety measurement, safety monitoring, prehospital

## Abstract

**Background:**

Prehospital care is potentially hazardous with the possibility for patients to experience an adverse event. However, as compared to secondary care, little is known about how patient safety is managed in prehospital care settings.

**Objectives:**

The objectives of this systematic review were to identify and classify the methods of measuring and monitoring patient safety that have been used in prehospital care using the five dimensions of the Measuring and Monitoring Safety (MMS) framework and use this classification to identify where there are safety ‘blind spots’ and make recommendations for how these deficits could be addressed.

**Methods:**

Searches were conducted in January 2020, with no limit on publication year, using Medline, PsycInfo, CINAHL, Web of Science and Academic Search. Reference lists of included studies and existing related reviews were also screened. English-language, peer-reviewed studies concerned with measuring and monitoring safety in prehospital care were included. Two researchers independently extracted data from studies and applied a quality appraisal tool (the Quality Assessment Tool for Studies with Diverse Designs).

**Results:**

A total of 5301 studies were screened, with 52 included in the review. A total of 73% (38/52) of the studies assessed past harm, 25% (13/52) the reliability of safety critical processes, 1.9% (1/52) sensitivity to operations, 38.5% (20/52) anticipation and preparedness and 5.8% (3/52) integration and learning. A total of 67 methods for measuring and monitoring safety were used across the included studies. Of these methods, 38.8% (26/67) were surveys, 29.9% (20/67) were patient records reviews, 14.9% (10/67) were incident reporting systems, 11.9% (8/67) were interviews or focus groups and 4.5% (3/67) were checklists.

**Conclusions:**

There is no single method of measuring and monitoring safety in prehospital care. Arguably, most safety monitoring systems have evolved, rather than been designed. This leads to safety blind spots in which information is lacking, as well as to redundancy and duplication of effort. It is suggested that the findings from this systematic review, informed by the MMS framework, can provide a structure for critically thinking about how safety is being measured and monitored in prehospital care. This will support the design of a safety surveillance system that provides a comprehensive understanding of what is being done well, where improvements should be made and whether safety interventions have had the desired effect.

## Introduction

Over recent decades, prehospital care has evolved from a transportation organization model to an integrated part of the healthcare system where advanced care is provided to critically ill and injured patients [1]. Prehospital care is potentially hazardous with the possibility for patients to experience an adverse event. However, as compared to secondary care, little is known about how patient safety is managed in prehospital care settings [1].

This lack of reliable data on safety is a common problem across healthcare. In order to address the established deficiencies in measuring and monitoring patient safety, Vincent *et al*. [2, 3] developed the Measuring and Monitoring Safety (MMS) framework. The MMS framework includes five dimensions that should be covered in any safety monitoring approach in order to give a complete and comprehensive understanding of an organization’s safety. A description of these dimensions is provided in Table 1.

**Table 1:** Description of the five dimensions of safety (adapted from Vincent *et al.* [3])

MSS dimension	Purpose	Examples
Past harm: Has patient care been safe in the past?	Assess rates of past harm to patients.	Mortality and morbidity Patient record review
Reliability: Are clinical systems and processes reliable?	Assess the reliability of safety critical processes and the ability of staff to follow these procedures.	Monitoring of vital signs Observations of safety critical behaviour Audit of equipment availability
Sensitivity to operations: Is care safe today?	Support the monitoring of safety on an hourly or daily basis.	Observations and conversations with clinical teams Talking to patients Briefings and debriefings
Anticipation and preparedness: Will care be safe in the future?	Identify future threats to safety.	Safety climate assessment Structured reflection Human reliability analysis
Integrating and learning: Are we responding and improving?	Analysis and use safety information to improve safety.	Aggregate data on patient complaints Feedback and implementation of safety lessons

Given the limited focus on prehospital care in the patient safety literature, the aims of the systematic review reported in this paper are to identify and classify the methods of measuring and monitoring patient safety that have been used in prehospital care using the five dimensions of the MMS framework and use this classification to identify where there are safety ‘blind spots’ and make recommendations for how these deficits could be addressed.

## Methods

This review is reported in accordance with the Preferred Reporting Items for Systematic Reviews and Meta-Analyses (PRISMA) guidelines [4].

### Search strategy

Systematic searches were conducted in January 2020 of five electronic databases: Medline, PsycInfo, CINAHL, Web of Science and Academic Search Complete. The search strategy (for sample Medline search strategy, see Online Supplementary Materials 1) included Medical Subject Headings terms along with free-text keywords, and the strategy was altered as necessary for databases other than Medline. No limits were placed on publication year.

The returns within each database were screened by manuscript authors R.O.M. or A.M.O. They reviewed each title and abstract and considered these in relation to the review’s inclusion and exclusion criteria. For returns that appeared eligible for inclusion, or returns in which the title and abstract did not provide sufficient information for the determination to be made, the full text of the paper was accessed. All full texts were reviewed by the research team together who discussed each paper and made a final decision regarding inclusion or exclusion of each text. Finally, the reference lists of all studies identified for inclusion from the electronic searches were screened in order to identify any other articles that were potentially suitable for inclusion. In addition, the reference lists of two other recent reviews pertaining to patient safety in prehospital care settings were also examined [5, 6].

### Study selection

#### Inclusion Criteria

In order to be eligible for inclusion in this review, studies had to be published in a peer-reviewed journal; be concerned with measuring and monitoring safety in prehospital care; report original research; evaluate a tool or measure for assessing attitudes towards, or engagement regarding, patient safety in a prehospital care setting and written in English.

#### Exclusion Criteria

Studies were excluded if they were focused on patient safety among those patients with a specific medical condition or those taking a specific medication; the measurement of participants’ perceptions of specific types of patient safety tools; one particular prehospital function or process (e.g. prescribing); a healthcare setting other than prehospital; safety data collected from multiple settings, including prehospital care, where prehospital data cannot be extracted for the main outcome measure; intra- and inter-hospital transport/transfer of patients within the hospital or by a specialized team; the measurement of provider safety and injury only; measurement tools that cannot be used in practice or that focus on perceptions/opinions of hypothetical scenarios and the measurement of patient safety in a simulated environment.

### Data extraction

A structured tool was used to extract information from each included study on the tool(s) name; type of tool(s); description of tool(s); country in which the study was conducted; type of study; clinical application of the tool(s) and the number of applications of the tool(s). Data extraction was conducted independently by three of the authors (A.M.O., K.L. and R.O.M.), with two of these authors extracting data independently for each of the included articles. Disagreements between reviewers were resolved through discussion until consensus was achieved.

### Quality assessment

Two reviewers (either R.O.M., K.L. or A.M.O.) critically appraised each of the included studies using the Quality Assessment Tool for Studies with Diverse Designs (QATSDD) [7]. The QATSDD allows for the methodological assessment of studies using quantitative, qualitative and mixed-method research designs. Scores on this measure can range from 0 to 46 for quantitative and qualitative studies and from 0 to 48 for mixed-method studies. Reviewers completed the quality assessment of each study by working together, and disagreements were resolved through discussion.

### Data synthesis

The methods of measuring and monitoring safety used in each study were categorized using the five domains described in the MMS framework shown in Table 1. A study may use more than one method, and some methods may be categorized under more than one dimension. The categorization was carried out by consensus between three reviewers (R.O.M., K.L. and A.M.O.).

## Results

**Figure 1:**
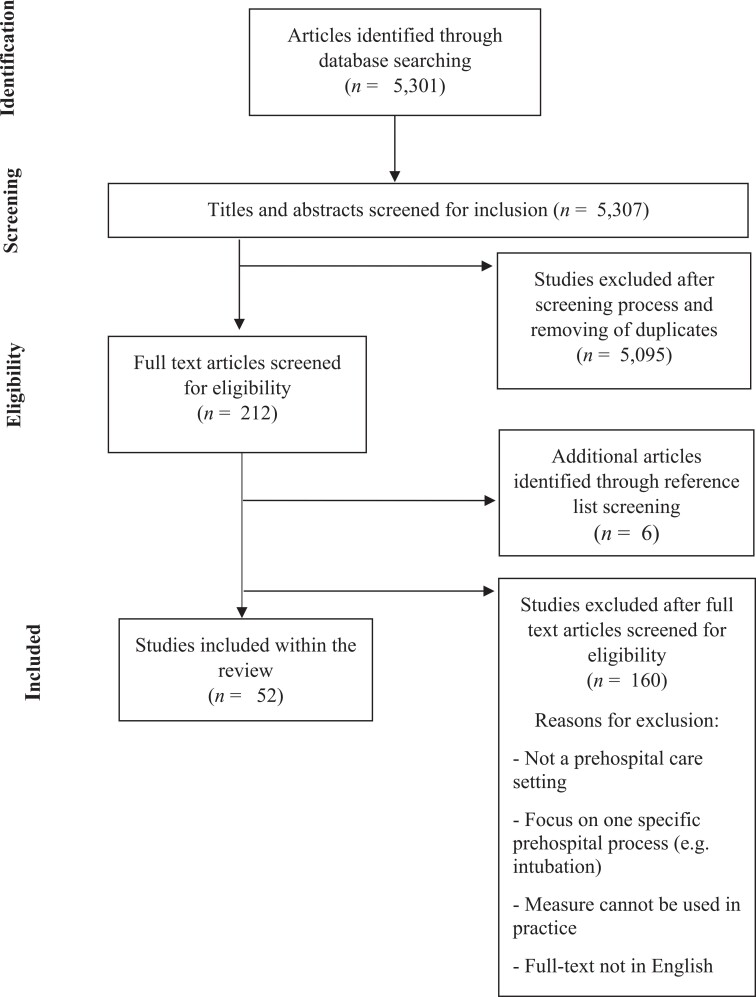
PRISMA flow diagram.

In total, 5301 articles were retrieved in the electronic database search (see Figure 1). A total of 212 papers were retrieved for full-text screening, of which 46 studies ultimately met the inclusion criteria. Six additional studies were identified through reference list and bibliography screening, bringing the total number of included papers to 52 [8–59]. Across these studies, 67 assessments of patient safety in prehospital care settings were described. A comprehensive summary of each study is presented in Online Supplementary Materials 2.

### Study characteristics

The 52 included studies were published between 1991 and 2019. Half (26/52) of the studies were conducted in North America, 30.8% (16/52) in Europe, 13.5% (7/52) in Australia and 5.8% (3/52) in Asia.

### Methods of measuring and monitoring safety

The 52 studies included in the review used 67 methods for measuring and monitoring safety. A total of 59.7% (40/52) of the studies reported an assessment of one of the MMS domains, 25.4% (17/52) reported an assessment at two of the MMS domains and 4.5% (3/52) carried out an assessment of three of the MMS domains. A total of 80.1% (42/52) studies included one method for measuring and monitoring safety, 11.5% (6/52) used two measures, 5.8% (3/52) used three measures and 1.9% (1/52) used four measures.

A total of 67 methods for measuring and monitoring safety were used in the included studies. Of these methods, 38.8% (26/67) were surveys, 29.9% (20/67) were patient record reviews, 14.9% (10/67) were incident reporting systems, 11.9% (8/67) were interviews or focus groups and 4.5% (3/67) were checklists.

### Past harm

Past harm was the most frequently assessed of the five MMS domains (73%; 38/52). Four different methods were used for measuring and monitoring past harm: patient record review, incident reporting systems, surveys and interviews/focus groups (see Table 2, and Online Supplementary Material 2).

**Table 2:** Summary of methods for measuring and monitoring patient safety

Methods for measuring and monitoring patient safety	Study references
Past harm (*n *= 38/52 studies; 73.1%)	
Patient record review (*n *= 21 studies)	
• The Emergency Medical Services Trigger Tool (*n* = 2)	[31, 32]
• Pittsburgh Adverse Event Detection and Classification Tool (*n* = 2)	[43, 44]
• Other trigger tools (*n *=* *17)	[16, 21, 23, 27, 28, 30, 35, 37–39, 45, 49–51, 55–57]
Incident reporting systems (*n* = 9 studies)	[19, 22, 25, 29, 40, 52, 55, 56, 59]
Surveys (*n *=* *8 studies)	
• The EMS Safety Inventory (EMS-SI) (*n* = 3)	[11, 46, 58]
• Other survey tools (*n* = 5)	[14, 15, 33, 34, 47]
Interviews/focus groups (*n* = 4 studies)	[12, 18, 19, 22]
Reliability of safety critical procedures (*n* = 13/52 studies; 25%)	
Patient record review (*n* = 1 study)	[51]
Surveys (*n *=* *11 studies)	
• The EMS Safety Inventory (EMS-SI) (*n* = 3)	[11, 46, 58]
• Other survey tools (*n* = 8)	[8, 9, 14, 17, 20, 24, 33, 34]
Practice assessment checklist (*n* = 1 study)	[36]
Interviews/focus groups (*n* = 1 study)	[40]
Sensitivity to operations (*n* = 1/52 studies; 1.9%)	
Interviews/focus groups (*n* = 1 study)	[10]
Anticipation and preparedness (*n* = 20/52 studies; 38.5%)	
Incident reporting systems (*n* = 1 study)	[29]
Surveys (*n *=* *15 studies)	
• The EMS Safety Attitudes Questionnaire (EMS-SAQ) (*n *=* *5)	[11, 13, 41, 42, 58]
• Norwegian Prehospital Survey of Patient Safety Culture (PreHSOPSC) (*n *=* *2)	[53, 54]
• Other survey tools (*n* = 8)	[8, 9, 15, 17, 20, 26, 33, 48]
Interviews/focus groups (*n* = 4 studies)	[10, 18, 19, 22]
Integration and learning (*n* =* *3/52 studies; 5.8%)	
Incident reporting systems (*n* = 3 studies)	[19, 22, 29]

### Reliability of safety critical processes

A total of 25% (13/52) of the included studies measured the reliability of safety critical processes in prehospital care (see Table 2 and Online Supplementary Material 2).

### Sensitivity to operations

Only one study included a measure that assessed the sensitivity to operations (see Table 2 and Online Supplementary Material 2). Atack and Maher [10] conducted interviews with Emergency Medical Service experts to develop an understanding of the current issues with patient safety (also categorized under the anticipation and preparedness domain).

### Anticipation and preparedness

Anticipation and preparedness was assessed in 38.5% (20/52) of studies (see Table 2 and Online Supplementary Material 2). The methods used included surveys, interviews or focus groups, and incident reporting systems.

### Integration and learning

Three studies employed measures of patient safety that corresponded to the domain of integration and learning (see Table 2 and Online Supplementary Material 2). The methods were focused on either the reporting or learning from patient safety incidents.

### Methodological rigour

The mean score on the QATSDD across all 52 studies was 25.40 (SD = 4.71; range = 16–36). QATSDD scores for each individual study are provided in Online Supplementary Material 2. Studies generally performed best on items relating to the selection of a suitable data collection tool, appropriate use of statistical tests and description of the aims and objectives of the study. However, studies tended to perform most poorly on items relating to the description of an explicit theoretical framework, involvement of the user in the design of the study and consideration of the sample size.

## Discussion

### Statement of principal findings

Past harm was the most frequently assessed of the five MMS domains, with patient record review the most frequently used approach. A quarter of the included studies measured the reliability of safety critical processes. Only one study included a measure that assessed the sensitivity to operations. Anticipation and preparedness was assessed in just over a third of the included studies. Integration and learning was assessed in only three of the included studies.

### Strengths and limitations

The strengths of this review are the use of an appropriate systematic review methodology, transparent inclusion/exclusion criteria, a comprehensive search across five key electronic databases and the presentation of the findings using the PRISMA reporting. There are also a number of limitations of this review. First, restricting the searches to English language articles may have resulted in the omission of methods of measuring and monitoring safety. Second, searches of the grey literature were not carried out and only peer-reviewed publications were included. However, there are issues with including grey literature searches within a systematic review such as compromised methodological reproducibility and difficulties in interpreting these publications due to the low methodological quality and poor reporting [60]. Third, we assessed the quality of studies using the QATSDD. The QATSDD has been assessed by its authors for reliability and validity and found to be acceptable [7]. However, the evaluation is subjective and concerns have been raised about the tool’s structure, particularly around the equal weighting of all items for all studies [61].

### Interpretation within the context of the wider literature

Almost three quarters of the included studies incorporated a measure of past harm, typically patient record review. Patient record review is often regarded as the ‘gold standard’ research method in patient safety [62]. However, patient record reviews attempt to address a very broad range of incidents and types of harm [2, 3, 62, 63]. Such a broad review fails to support the detection of important differences in safety between different prehospital care providers or allow comparisons across time. Therefore, the authors recommend that prehospital care researchers and practitioners should carry out patient record reviews to identify the prevalence of specific safety events of interest (e.g. medication error) or errors associated with specific tasks (e.g. airway insertion) in order to identify areas in which patient safety could be improved.

The reliability of safety critical processes and systems was assessed in a quarter of papers included in the systematic review. Reliability can most readily be applied to standard aspects of healthcare delivery that should happen with every patient (e.g. monitor vital signs [3]). In secondary care, reliability tends to be assessed through clinical audit. However, our review found that in prehospital care, self-report survey was the most common method of assessing reliability. A reliance on self-report is problematic as there is a tendency to overestimate the reporting of desirable behaviour [64]. Therefore, there is a need to consider more objective approaches to measuring reliability in prehospital care. This objectivity could be achieved through the use of technology. A recent pilot study found that body-worn cameras were an effective means of identifying errors in documentation by Emergency Medical Technicians following a simulated patient encounter [65]. Moreover, it is becoming increasingly common for patient monitors to record and store cardiopulmonary resuscitation data (e.g. depth of chest compressions [66]). Therefore, the authors suggest that there is a need to explore the feasibility of using technology to collect data on the reliability of safety critical data, rather than relying on self-report.

Only one included study examined whether care was safe today (sensitivity to operations). This study used interviews with staff to identify current patient safety issues [10]. It is also suggested that input from patients is also a valuable, and often under-utilized, source of safety information [3]. Patients and their families have privileged access to information on continuity of care, communication failures and dignity issues [67]. Also, patients and their families are outside the healthcare organization and so provide an independent assessment of that organization. It is recognized that it may be more challenging to obtain patient feedback on prehospital care as compared to secondary care. However, there are existing mechanisms that could be adapted to collect data on safety in prehospital care (e.g. adding specific questions on prehospital care to patient experience surveys). The authors suggest that organizations and researchers consider how to obtain information from patients on their experience in prehospital care settings.

Just over a third of the included papers considered whether patients would be safe in the future (anticipation and preparedness). Future safety is important for strategic planning, and it has been acknowledged that this is something that is under-developed in healthcare [3]. Anticipation and preparedness were most commonly assessed using surveys. Surveys are attractive as they allow a large amount of data to be collected with little effort. However, there are limitations such as low response rates and other sources of measurement error that negatively impact the validity and usefulness of survey data [68]. Moreover, even valid and reliable surveys only provide a broad overview of attitudes of staff to safety. They provide little detailed information on specific safety issues, nor do they provide reasons why particular safety issues may exist. This is not to say that useful data do not come from surveys. However the authors recommend that surveys should be used as part of a larger organizational safety surveillance programme. The authors further suggest that efforts should be made to combine surveys with other qualitative approaches, such as interviews or focus groups, in order to contextualize the survey findings.

Only three studies considered whether the organization were responding to issues and improving safety (the integration and learning domain). The small number of studies assessing this domain may seem surprising. However, this deficiency is common across healthcare, and many high-profile enquiries into healthcare scandals have identified a failure for healthcare organizations to learn from safety data (e.g. Mid Staffordshire NHS Foundation Trust Public Inquiry). Certainly, learning from poor performance is important (known as a Safety I approach [69]). However, in recent years, it has been suggested that there is merit from not only studying the uncommon examples of deficient performance, but also to study why care is delivered safely. Unsafe care accounts for only a tiny minority of the activities carried out in prehospital care. Learning from why things ‘go right’ (known as taking a Safety II approach [69]) is an important and under-utilized source of safety information. The authors suggest that efforts should be made to identify how to learn from not only the rare examples of unsafe care in prehospital settings, but also from safe care.

### Implications for policy, practice and research

A single measure of safety is a fantasy [3], with every method of measuring and monitoring safety having both strengths and limitations. A robust safety surveillance system must include multiple methods that cover all five domains of the MMS framework. Mapping existing methods of measuring and monitoring safety against the MMS framework allows organizations not only to consider where information is lacking, but also where there is redundancy and duplication of effort. It has been suggested that healthcare stakeholders could get the information they need with 25% of what is currently being spent on measurement [70]. Therefore, the aim of a safety surveillance system should be to measure only what matters and focus on learning [70], rather than on quantity of data and satisfaction of mandatory reporting requirements. Measures that are too burdensome or lack credibility may alienate staff and lead to confusion about the impact of interventions [71]. Therefore, organizations should review existing safety monitoring systems in order to identify blind spots as well as where there may be duplication of effort.

It is also important that safety data are readily interpretable by both frontline staff and management so that safety issues can be identified and addressed. It can be challenging to integrate data from multiple measures of safety. In secondary care settings, hospital-wide safety dashboards have been developed to help present and integrate safety data from multiple sources. These information presentation systems allow any safety issues to be identified, allow for a comparison to be made between organizations and help determine whether interventions to improve safety have had the desired effects [72]. There is a need to develop safety dashboards to provide meaningful and readily interpretable safety data for prehospital care settings.

## Conclusions

There is no single method of measuring and monitoring safety in prehospital care, with every approach having both strengths and limitations. Arguably, most safety monitoring systems have evolved, rather than been designed. This leads to safety blind spots in which information is lacking, as well as to redundancy and duplication of effort. It is suggested that the findings from this systematic review, informed by the MMS framework, can provide organizations and researchers with a structure for critically thinking about how safety is currently being measured and monitored in prehospital care settings. This will support the design of a safety surveillance system that provides a comprehensive understanding of what is being done well, where improvements should be made and whether safety interventions have had the desired effect.

## Supplementary Material

mzab013_Supp

## Data Availability

All data is either presented in the article, or the included supplemental material.
