# Supplementary material for: Measurement and monitoring patient safety in prehospital care: a systematic review
Source: Int J Qual Health Care. 2021 Jan 18;33(1):mzab013. doi: 10.1093/intqhc/mzab013 (PMC10517741; doi:10.1093/intqhc/mzab013)
Supplement: mzab013_Supp [file mzab013_supp.zip › suppl_data/Supplementary material 1 - search strategy.docx]

**Appendix 1**

**Summary of Medline (OVID) Search Strategy**

1. exp Emergency Medical Service/

2. exp Ambulances/

3. Pre?hospital.ti,ab.

4. out-of-hospital*.ti,ab.

5. (emergency adj1 care).ti,ab.

6. (emergency adj1 service*).ti,ab.

7. exp Emergency Medical Technicians/

8. (emergency adj2 technician*).ti,ab.

9. paramedic*.ti,ab.

10. (emergency adj2 transport*).ti,ab

11. (patient* adj1 transport*).ti,ab.

12. (patient* adj1 transfer*).ti,ab.

13. (int*facility adj1 trans*).ti,ab.

14. (int*hospital adj1 trans*).ti,ab.

15. Medical evacuation.ti,ab.

16. Med?vac*.ti,ab.

17. OR/1-16

18. exp Patient Safety/

19. (patient* adj1 safe*).ti,ab.

20. (safe* adj1 climate*).ti,ab.

21. (safe* adj1 culture*).ti,ab.

22. (safe* adj1 attitude*).ti,ab.

23. (safe* adj2 behav*).ti,ab.

24. (patient* adj2 harm).ti,ab.

25. (adverse adj event*).ti,ab.

26. (sentinel adj event*).ti,ab.

27. exp Medical error/

28. (health?care adj error*).ti,ab.

29. (medica* adj error*).ti,ab.

30. malpractice.ti,ab.

31. (near miss*).ti,ab.

32. OR/18-31

33. survey*.ti,ab.

34. questionnaire*.ti,ab.

35. measure*.ti,ab.

36. instrument*.ti,ab.

37. tool*.ti,ab.

38. interview*.ti,ab.

39. checklist*.ti,ab.

40. report.ti,ab.

41. OR/33-40

42. 17 AND 32 AND 41
