# Supplementary material for: Measurement and monitoring patient safety in prehospital care: a systematic review
Source: Int J Qual Health Care. 2021 Jan 18;33(1):mzab013. doi: 10.1093/intqhc/mzab013 (PMC10517741; doi:10.1093/intqhc/mzab013)
Supplement: mzab013_Supp [file mzab013_supp.zip › suppl_data/Supplementary material 2 - summary of studies.docx]

**Appendix 2**

Table 3. Summary of measurement tools used across studies and their corresponding safety domain

| **Study** | **Tool Format**  **Tool Name (if any)** | **Research Design** | **Detailed Tool Description** | **Country** | **Clinical Setting** | **No. of Applications** | **Safety Domain** | **Study Quality Score** |
| --- | --- | --- | --- | --- | --- | --- | --- | --- |
| Albertsson et al. (8) | Staff survey | Quasi-experimental study | The self-assessment questionnaire used was a 24-item measure of   - ambulance drivers’ self-assessed driving performance - driving competence - driving style - self-reflection - driving safety attitudes. | Sweden | Ambulance drivers at two ambulance stations in Northern Sweden | 74 applications at pre-test and 74 applications at post-test | Self-assessment  - Anticipation and preparedness.  - Reliability | 22 |
| Andel et al. (9) | Staff survey  National Institute for Occupational Safety and Health (NIOSH) short Safety Climate Scale  Staff survey  Staff survey | Three wave longitudinal survey | NIOSH – 6-item survey assessing safety climate perceptions.  -responses are rated on a four-point Likert scale ranging from *strongly disagree* to *strongly agree.*  Safety Compliance staff survey – 3-item survey developed by Neal and Griffin (2004) measuring safety compliance.  -responses are rated on a five-point Likert scale ranging from *strongly disagree* to *strongly agree.*  Safety Participation staff survey – 3-item survey developed by Neal and Griffin (2004) measuring safety participation.  -responses are rated on a five-point Likert scale ranging from *strongly disagree* to *strongly agree.* | USA | Publicly available list of licensed EMS professionals on the Florida Department of Health website. | NIOSH -335 at Time 1  Safety Compliance survey – 298  -166 at Time 2  &  -132 at Time 3  Safety Participation survey – 298  -166 at Time 2  &  -132 at Time 3 | NIOSH  - Anticipation and preparedness.  Safety Compliance survey  - Reliability  Safety Participation survey  - Reliability  - Anticipation and preparedness. | 28 |
| Atack et al. (10) | Staff interview | Qualitative cross-sectional study | In-depth 1:1 interviews were conducted with informants to gain an in-depth understanding of major issues pertaining to EMS patient safety.  Avg 40 minutes  Informants were first asked two broad opening questions that allowed the informants to raise topics they deemed relevant and important for discussion:   - “What do you see as the most important issues regarding patient safety in EMS today?”, and - “What factors in the EMS environment might adversely affect patient safety?”   Informants were then asked to:   - Comment on two safety issues; medication errors and vehicle accidents, and - Discuss knowledge gaps, implications for practice, future research, and recommendations for change. | Canada (two participants from UA and Europe respectively) | Purposive sample of informants with knowledge and expertise regarding policy, practice and research who could speak to the issue of patient safety: paramedics, emergency physicians, EMS researchers and administrators, plus patient representative from Canada, the USA, and Europe | 16 informants (14 from Canada, 1 from USA, and 1 from Europe) | Staff Interview  - Sensitivity to operations  - Anticipation and preparedness | 29 |
| Baier et al. (11) | Staff survey  The EMS Safety Inventory (EMS-SI)  Staff survey  The EMS Safety Attitudes Questionnaire (EMS-SAQ). | Cross-sectional study | EMS-SI - 44-item survey that measures self-reported safety outcomes from EMS workers.  Items grouped into three composite measures  Two nominal, seven-option, categorical scales used.   - provider injury (2 items) - patient care error or AE (25 items) - safety-compromising behaviours (17 items)   EMS-Safety Attitudes Questionnaire (EMS-SAQ)  60-item survey assessing patient safety culture (Not all items considered in this study)  Items scored on 5-point Likert scale  Six domains:   - safety climate - teamwork climate - perceptions of management - working conditions - stress recognition - job satisfaction | Germany | EMS-workers in Germany, recruited via social media channels and the professional association for non-medical EMS-workers | 1101 questionnaires | EMS-SI  - Past harm  - Reliability  EMS-SAQ  - Anticipation and preparedness | 25 |
| Benneck et al. (12) | Staff interview | Cross-sectional | Face-to-face interviews based on the critical incident technique (CIT) were used to explore registered nurses-experiences and behaviours associated with near misses where patient safety in the ambulance service was jeopardised.  15-30 minutes each  The interviews started with an open request:   - “Please, describe an incident at work when the safety of the patient was jeopardized.”   This request was followed by open, in-depth follow-up questions such as “How do you mean?” and “Please, explain more.” | Sweden | Registered nurses working in one ambulance service organisation in mid-Sweden. The regional organization with 8  ambulance stations had 13 ALS ambulances around the clock and 5  daytime ALS ambulances | 15 informants | Staff Interview  - Past harm | 29 |
| Bitan et al. (13) | Staff survey  The EMS Safety Attitudes Questionnaire (EMS-SAQ) | Two-wave longitudinal survey (unpaired) | EMS-SAQ – 43-items from the 60-item EMS-SAQ were employed to assess patient safety culture, under 6 domains:   - safety climate - job satisfaction - perceptions of management - teamwork climate - working conditions - stress recognition   Responses to EMS-SAQ items are captured on a five-point Likert scale (strongly disagree; disagree; neutral; agree; strongly agree). | Canada | Paramedics from six pre-hospital emergency care services operating under one regional hospital program in Ontario, Canada | 2082  (1035 in 2014 and 1047 in 2015) | EMS-SAQ  - Anticipation and preparedness. | 27 |
| Blau et al. (14) | Staff survey  The Haddon Matrix | Cross-sectional study | The Haddox Matrix  A 12-cell matrix was used to understand EMS provider and patient injury prevention and safety during ambulance response and transport.  The four general components of the Haddon Matrix containing variables that contribute to an injury event are the: host (person(s) at risk), agent (equipment that contributes to an injury), physical environment (the setting characteristics where the injury  occurs) and social environment (the legal and cultural norms and  practices in society).  The second dimension of the Haddon Matrix gives information about the timing of factors in an injury event occurrence: pre-event, event and post-event.  These four (general) by three (time) components create a 12-cell matrix within which specific variables are noted.  Pre-event host variables   - Gender (1 item) - Highest education level (1 item) - General perceived health (2 items) - EMT experience in years (1 item) - Extrinsic satisfaction (5 items) - Intrinsic satisfaction (5 items) - Prior ambulance accident (1 item; rated as Yes/No question)   Event host variables   - Time in patient compartment (1 item) - Seatbelt use (2 items; rated on 5-point Likert scale)   Safety Outcomes – three safety variables were measured:  - Anticipated safety equipment use (3 items)   - Restraints that allow for mobility - Oxygen bottle mounting system on stretcher - Helmet with integrated communication system   - Patient secure (2 items)   - Patient to stretcher - Stretcher   - Equipment secure (6 items)   - Defibrillator - Portable oxygen cylinder - Airway bag - Trauma bag - Medication bag - Portable suction unit   Responses to all three measures are captured on a five-point Likert scale ranging from *never* to *all the time*. | USA | Nationally certified EMS professionals registered with the US National Registry of Emergency Medical Technicians. Sample based on responses to annual distribution of Longitudinal Emergency Medical Technician Attributes and Demographic Study. | 648 EMS professionals | The Haddox Matrix  (Prior ambulance accident)  - Past harm  - Reliability | 24 |
| Boal et al. (15) | Staff survey | Cross-sectional study | For each of the five routes of exposure, respondents were asked how many times they had contact with blood during the previous 12 months.  The five routes included:   1. needle or lancet stick after use on a patient 2. cut by scalpel, razor, scissors, or sharp object like glass or metal that had blood or body fluid containing visible blood on it 3. blood or body fluid containing visible blood in the eyes, nose, or mouth (hereafter referred to as mucous membrane exposures) 4. blood or body fluid containing visible blood on non-intact skin 5. human bite   Questions included   - # contacts with blood during previous 12 months for each route - Additional questions about two most recent exposures for each route of exposure, including whether exposure was reported to relevant authorities. Four possible reasons for non-reporting offered:   - It wasn’t a significant exposure   - I didn’t want to be reprimanded   - Confidentiality   - Other (specify) - Whether respondent would report exposures under specified conditions (5-point Likert scales) - Attitude towards exposure and prevention (5-point Likert scales)   Two questions using a yes/no/don’t know format were  included as indicators of management emphasis on workplace safety culture.  These were:   - If you did not follow Universal/Standard Precautions, would your supervisor speak to you about it? - Is following safety procedures part of your job evaluation? | USA | Nationally representative sample of 5000 paramedics in USA plus simple random sample of 1500 paramedics in California (due to California state needlestick prevention laws)  Eligible paramedics with direct patient contact and at least 4 calls in previous 4 weeks | 2,664 paramedics | Blood exposure  - Past harm  - Anticipation and preparedness | 23 |
| Boyle et al. (16) | Retrospective review of preventable death study reports | Retrospective review of reported errors | A retrospective review of reported prehospital errors in six preventable death study reports published by the Consultative Committee on Road Traffic Fatalities (CCRTF) was conducted to identify and interpret prehospital error rate trends associated with road traffic fatalities over a 10-year period of the CCRTF reports.  Error were categorised under five categories:   - system inadequacy - error in treatment/management strategy - error technique - error in diagnosis - delay in diagnosis   Two multidisciplinary medical groups reviewed a range of medical documents (prehospital through to autopsy reports) to assist them in determining the errors, including those contributing to death. | Australia | Six reports by Consultative Committee on Road Traffic Fatalities covering ten years 1994-2003, in Prehospital services in Victoria, Australia | 843 fatalities total | Respective review  - Past harm | 20 |
| Chesters et al. (17) | Staff survey  Helicopter Emergency Medical Services - Safety  Survey | Cross-sectional study | The 74-item HEMS-SS was used to describe and compare the attitudes and perceptions towards risk in HEMS operations of these staff.   - Perception of inherent safety of HEMS operations (binary yes/no), - the likelihood of various factors increasing or reducing the risk of a HEMS crash or serious incident, with responses measured on a 5-point Likert scale, - risk of crash or serious incident at various times of journey (Primary/Scene calls and Secondary/inter-hospital/inter-facility transfers), rated on a ranking scale (scale 1-6), - assessment of contributory factors to crash depicted in fictional scenario (scale 1-10), - local reporting and safety activities, - previous training received, - staff perceptions of aviaton risk, - staff ability to contribute to decisions, and - dispatch criteria and aircraft utilisation | UK | Purposive sample of key personnel at each of the air ambulance services in the UK and two large organisations providing air ambulance services in the UK, plus members of The Association of Air Ambulances | 100 survey responses | HEMS-SS  - Anticipation and preparedness.  (the overall survey)  - Reliability | 26 |
| Cottrell et al. (18) | Staff focus groups | Cross-sectional focus group study | Staff focus groups were conducted to understand  (1) patient safety issues that occur in the prehospital care of children, and  (2) factors that contribute to these safety issues (e.g., patient, family, systems, environmental, or individual provider factors).  Typically had 8-12 participants and ran for approximately 60-90 minutes.  Opened with accepted definition of safety events (adverse events, near misses, errors)  The survey provided a list of factors that could potentially  contribute to a crash and respondents were asked to rate them  from 1 (very likely to contribute to a crash) to 5 (very unlikely  to contribute to a crash).  The focus group discussions were guided by five questions:   1. What are the most challenging calls involving children? 2. Can you think about a specific example of a safety event or a near miss involving a paediatric patient? 3. Are there particular situations that you think increase the risk of safety events in paediatric patients? 4. What are the most important factors contributing to safety events? 5. Do you feel comfortable reporting errors or near misses that you have made? How about those you have observed? | USA | EMS providers, incl basic, intermediate and paramedic EMTs of all levels in both rural and urban settings in a densely populated (>700,00) urban/suburban county of Oregon, USA. | 40 informants (4 focus groups of 8-12 participants) | Staff focus groups  - Past harm  - Anticipation and preparedness | 32 |
| Cushman et al. (19) | Staff event reporting system  Staff semi-structured interviews  Staff focus groups | Cross-sectional qualitative study | Complementary qualitative data-collection methods  of an event reporting system, semi-structured interviews, and focus groups were used to identify emergency medical services (EMS) provider perceptions of factors that may affect the occurrence, identification, reporting, and reduction of near misses and adverse events in the paediatric EMS patient.  Staff event reporting system -  EMS providers could access an anonymous event reporting system online. Electronic reports included the following information:   - event classification (near miss or adverse event), - reporter level of emergency medical technician (EMT) certification, - setting of incident (career or volunteer agency), - impact on patient, - description of the event, and - response and repercussions   Semi-structured interviews –  Semi-structured interviews were conducted to assess ambulance personnel’s’ perceptions of near misses and adverse events in paediatric patients. A semi-structured guide was followed and probing questions were used to gather detail and to further explore responses related to child health emergencies.  Purposive sampling to identify a broad range of individuals (EMS providers at all levels, volunteer and paid services, range of certification levels, varied levels of experience).  Interview schedule included   - Definition of adverse event, medical error, near miss - Description of situations in which these were witnessed in paediatric and adult patients - Description of situation surrounding the event - Contributing factors to the event - Event reporting   Focus groups –  Focus groups were conducted to examine the nature of near misses and adverse events in EMS. Specific adverse event reports were not solicited because of the nonconfidential nature of the focus group; however, probing and follow-up questions were used to gather  detail and to explore responses.  Focus group schedule included   - Definitions of adverse event, medical error, near miss, and discussions of definitions - Situations in which these were witnessed and environments that lead to these - Scenario and what could have been done differently - Ideas for preventing future incidents - Who can be contacted to talk about a call - Events that would / wouldn’t be reported - Sufficient opportunity to report - Use of anonymous reporting, why / why not - Helpful to learn from others’ mistakes - Surprising or helpful content from group discussion - Desired improvements to EMS system for patient safety | USA | EMS providers from one of 40 volunteer and career EMS agencies in a two-county region in Western New York, USA. | 11  anonymous Web-based reports  17 semi-structured interviews,  Two focus groups with a total of 23 participants | Anonymous events reporting system  - Past harm  - Integration and learning  Semi-structured interviews  - Past harm  - Anticipation and preparedness  Focus groups  - Past harm  - Anticipation and preparedness | 28 |
| Eliseo et al. (20) | Staff survey  Staff survey | Cross-sectional study | Safety compliance scale – A 9-item list of safety protocols was used to assess adherence to safe work practices (i.e., universal precautions).  Adapted from an instrument assessing self-reported compliance with universal precautions by Gershon et al   1. Dispose of sharp objects in sharps container 2. Follow universal precautions 3. Wash hands after removing gloves 4. Wash hands whenever there is possible exposure 5. Wear eye protection whenever there is possible exposure 6. Recap contaminated needles 7. Encouraged to report all blood-borne exposures 8. Received training on exposure reporting 9. Reluctant to report exposures   *Strict adherence* was defined as answering 80% of items with a score of 4 or higher (i.e., “agree” or “strongly agree,” respectively) on a five-point Likert scale.  Safety climate scale – A 20-item instrument was used to assess perceived safety climate under 6 domains:   - availability of personal protective gear (2 items) - management support of safety-related programs (4 items) - lack of hindrances to safe work practices (3 items) - availability of frequent safety-related feedback and training (5 items) - tidiness of the workplace (3 items) - good communication/minimal conflict among co-workers (3 items) | USA | Full-time EMS providers in a two-tier (i.e., EMTs and paramedics) EMS system  in eastern Massachusetts, one of the busiest in the USA. | 196 EMS workers | Safety compliance  - Reliability  Safety climate  - Anticipation and preparedness  -Reliability of safety critical processes | 32 |
| Eriksson et al. (21) | Staff chart review tool  the paediatric  prehospital safety event detection system  (PEDS) chart review tool | Retrospective chart review | PEDS Chart Review Tool – the 36-item chart review tool was used to identify paediatric prehospital adverse safety events with a median completion time of 7 mins.  The tools five section include:   - Case identification and clinical background (12 items) - Assessment, diagnosis, and clinical decision making (5 items) - Procedures and interventions (6 items) - Medications and fluids (11 items) - Overall assessment (2 items)   Events are also categorized by type (unintended injury or consequences, near misses, suboptimal actions, errors, or management complications), potential severity (mild, moderate, or severe), and preventability (10-point scale). | USA | EMS transports with lights and sirens (indicating a time-sensitive emergency condition such as cardiac arrest) from 2009 to 2011 for patients, 18 years of age in Multnomah County, Oregon. | 30 charts were reviewed | PEDS Chart Review Tool  - Past harm | 26 |
| Fairbanks et al. (22) | Staff event reporting system  Staff semi-structured interviews  Staff focus groups | Cross-sectional study | Triangulated ethnographic methods was used, including focus groups, interviews, and event reporting to examine the perceptions of  EMS providers regarding near misses and adverse events in out-of-hospital care and to explore potential factors that underlie such events, as well as providers’ responses to them.  Staff event reporting system –  An anonymous event reporting system was employed.  Electronic reports included the following information: Linked to popular EMS online discussion board for 6 months.  Fields included   - event classification (near miss or adverse event) - reporter level of emergency medical technician (EMT) certification - setting of incident (career or volunteer agency) - impact on patient - description of the event - response and repercussions   Staff semi-structured interviews –  Staff semi-structured interviews were conducted to assess EMS providers experiences of near misses and adverse events in EMS. Interviews performed by investigators with no medical training / affiliation with EMS.  The following interview guide was used:   1. How do you define an adverse event? Medical error? 2. Near-miss? (study definitions were then provided) 3. Can you describe some situations in which you witnessed adverse events, near-miss, or medical errors in paediatric patients? What about adult patients? 4. What was the situation surrounding the event? 5. What do you think caused the event? Were there any other contributing factors? 6. Did you report the event? To whom? 7. Do you have any further examples?   Follow-up probes were used to pursue lines of questioning and answers that developed.  Staff focus groups –  Staff focus groups were conducted to gain a further understanding of EMS providers’ perceptions regarding adverse events. One facilitator  Specific adverse event reports not solicited  Focus group facilitators used a standardized script designed to stimulate conversation and guide the discussion to topics uncovered in the interviews and event reporting.  The following focus group script was used:   1. In front of you are the definitions of an adverse event, medical error, and a near-miss. Do these definitions make sense and do they correspond with how you would define the terms? 2. Can you think of any situation in which you witnessed an adverse event, near-miss, or medical error? 3. What sorts of environments lead to these sorts of incidents? 4. I am going to describe an event that might happen in the field. When I am done I would like to hear ideas about how things could have been done differently, what caused the error, and who takes the most responsibility for the error occurring. 5. What are some ideas for preventing such events in the future? 6. What do you do when you want to discuss what happened on a call with someone? 7. What is an example of an event that would be reported? What is an example of one that would not? 8. Do you feel there is enough opportunity for disclosing errors? 9. Do you think that if there was an anonymous way to report adverse events you would use it? Why or why not? 10. Do you think it would be helpful for ALS providers to learn from other’s mistakes or just becoming aware of things that happened in the past? 11. Have you heard anything in this group discussion that surprised you or that you feel was helpful to you personally? 12. What improvements would you like to see in the EMS system to affect patient safety?   Follow-up questions were used extensively to pursue relevant information. | USA | EMS providers in a  North-eastern U.S. EMS region, serving in at least one of 40 volunteer and career  EMS agencies within a two-county EMS region including  urban, suburban, and rural populations. | 11  anonymous Web-based reports  15 semi-structured interviews  Two focus groups with a total of 23 participants | Anonymous events reporting system  - Past harm  - Integration and learning  Semi-structured interviews  - Past harm  -Anticipation and preparedness  Focus groups  - Past harm  - Anticipation and preparedness | 28 |
| Felzen et al. (23) | Retrospective case review protocol | Retrospective review of cases | Retrospective protocol analysis – Adverse event review  Adverse events were assessed in a 2-step process.  First, protocols were preselected if  (1) the free-text comments section contained parts of or the German words or word combinations for unsuccessful, instable, unstable, no sign of recovery, on-site physician, hypotensive, hypotension, allergy, allergic, anaphylaxis, anaphylactic, accident, accidental, erroneous, error, confusion, confused, and possible misspellings; or (2) the tele-EMS physician administered catecholamines (adrenaline, noradrenaline, and theodrenaline-cafedrine), antihistamines, or corticosteroids.  Second, these protocols were reviewed by two independent researchers, and disagreements were resolved by consensus. | Germany | Patients in primary emergency missions with consultation of the tele-EMS physician during the first 3 operational years (April 2014 to March 2017) of the prehospital telemedical emergency service, in the City of Aachen, Germany. | 6265 patients were analysed | Retrospective case review  - Past harm | 22 |
| Fournier et al. (24) | Staff survey of EMS programmes | Two-part study of Mobile Intensive Care Unit (MICU) safety | Staff survey of EMS programmes  Sections included:   - Internal design of the MICUs used by EMS programme, number and arrangement of seats in rear compartment, fixation methods of rear compartment material. To be completed by programme directors - Most common positioning of personnel in rear compartment, habits regarding their own safety and patient safety, how often seat belts were used in rear compartment. To be completed by as many personnel as possible in EMS programme. - Report own traffic collision experiences during last three years. | France | Survey distributed to 50 largest metropolitan EMS programmes in France  Mail survey, 49/50 responses  12 of 49 responses had at least 1 MICU accident;  6 out of those 12 suffered at least 1 death; | 49 EMS programmes | Survey  - Reliability of safety critical processes | 23 |
| Gallagher et al. (25) | Staff event reporting system  Pennsylvania EMS Safety Event Reporting System | Retrospective descriptive study | Pennsylvania EMS Safety Event Reporting System –  Each event report includes:   - the description of the event - the relationship of the reporter to the event - and the year in which the event occurred   Reports were assessed and placed into subjective categories that were determined after review of the report’s content.  These categories included:   1. Communications    1. Dispatch Issue    2. Nondispatch issue 2. Staffing or ambulance availability 3. Vehicle/transportation    1. Mechanical breakdown/maintenance    2. Driving issue or collision    3. Destination issue 4. Medical equipment    1. Equipment failure    2. Lack of needed equipment 5. Medication    1. Command orders outside of protocols    2. Wrong route    3. Storage, expiration, etc.    4. Look-alike 6. Actions/behavior    1. Training/clinical judgment    2. Nonmedical behavior    3. Patient behavior 7. Multiple patients 8. Accident scene management/scene safety 9. Medical procedure 10. Multiple agencies/multiple units/ALS vs. BLS issue 11. Protocol issue | USA | Incidents reported by EMS providers in Pennsylvania, USA.  All reports to statewide EMS safety event reporting system from inception (2003-2010) | 229 usable reports | Pennsylvania EMS Safety Event Reporting System  - Past harm | 21 |
| Gallego et al. (26) | Staff survey  Safety Attitudes Questionnaire (SAQ) | Cross-sectional | SAQ – 60-item survey assessing 6 domains of patient safety culture:   - Teamwork Climate (perceived quality of collaboration between staff) - Safety Climate (perception of strong organizational commitment to safety) - Job Satisfaction (positive feelings about work experience) - Stress Recognition (acknowledgement of the influence of stressors on work performance) - Perception of Management (approval of managerial action) - Working Conditions (perceived quality of work environment)   Respondents’ scores on these factor scales range from 1 (unfavourable) to 5 (favourable) attitudes. | Australia | All staff in the public health workforce in the state of South Australia (18 service types) including 2 prehospital services | 14,054 completed questionnaires | SAQ  - Anticipation and preparedness | 26 |
| Hagiwara et al. (27) | A Trigger tool | Retrospective record review | A trigger tool was used to  1) investigate the incidence of adverse events (AEs) in prehospital care, and  2) To investigate the factors contributing to AEs in prehospital care.  Eleven screening criteria were used for the medical record review:   - 1. Missing, incomplete, or unclear documentation for the following: chief complaint, physical assessment, vital signs, hemodynamic monitoring (e.g., ETC02), allergies, pertinent history or medications, patient condition at handoff of facility   2. Time from initial patient contact to transfer of care exceeds accepted standards   3. Injury to patient or team member during patient encounter/transport (e.g., stretcher drop, needle stick, or other)   4. Request for additional resources, personnel, or supervisor due to change in patient condition   5. A worsening trend (deterioration) in patient hemodynamic or mental status indicators (e.g., vital signs, level of consciousness Glasgow Coma Scale score)   6. Cardiac arrest during transport   7. Use of any of the following interventions: cardioversion, defibrillation, transcutaneous pacing, advanced airway attempt, surgical airway, intraosseous access, chest decompression, chest tube 8. Failure of any intervention or procedure during patient care (some examples include: inability to obtain vascular access after a reasonable amount of time or number of attempts, failed intraosseous access, failed nasogastric tube placement, failed Foley placement, failed cardioversion, failed defibrillation, failed transcutaneous pacing, failed advanced airway or rescue airway, failed surgical airway, failed chest decompression)   8. Use of following medications or fluids: (blood products, vasopressors or inotropes [e.g., dobutamine, dopamine], naloxone, rapid sequence intubation medications [e.g., succinylcholine])   9. Evidence suggestive of deviation from standard of care by performing an intervention or administering a medication that appears to be outside protocol or failure to perform an intervention or provide a medication that is within the standard of care   10. Medication error (e.g., administering wrong or unapproved dose, administering wrong or unapproved medication, administering medication via wrong or unapproved route.   If any triggers were found, they were classified by severity and nature of the error was profiled. | Sweden | Ambulance personnel in three prehospital organisations; one urban environment, one mixed environment, and one rural environment. | 1080 medical records were included | Trigger tool  - Past harm | 31 |
| Hagiwara et al. (28) | A Trigger tool | Retrospective medical record review | Medical record review trigger tool –  Ambulance medical record were screened according to eleven screening criteria:  Documentation triggers   1. Missing, incomplete, or unclear documentation for the following: chief complaint, physical assessment, vital signs, haemodynamic monitoring, allergies, pertinent history or medications, patient condition at handoff.   Operational & patient movement triggers   1. Time from initial patient contact to transfer of care exceeds accepted standards. 2. Injury to patient or team member during patient encounter/transport. 3. Request for additional resources, personnel, or supervisor due to change in patient condition.   Patient condition triggers   1. A worsening trend in patient haemodynamic or mental status indicators. 2. Cardiac arrest during transport. Intervention & medication triggers 3. Use of any of the following interventions during patient care: cardioversion, defibrillation, transcutaneous pacing, advanced airway attempt, surgical airway, intraosseous (IO), chest decompression, chest tube. 4. Failure of any intervention or procedure during patient care. 5. Use of following medications or fluids: blood products, vasopressors, inotrope, naloxone. 6. Evidence of deviation from standard of care by performing an intervention or administering a medication that appears to be outside protocol or failure to perform an intervention or provide a medication that is within the standard of care. 7. Medication error.   Identified triggers were classified under five different categories:   - Actions By Patient: The adverse event was the result of action(s) by the patient. - Actions By Provider: The adverse event was the result of action(s) or inaction(s) by the crew. - Medical or Vehicle Equipment: Failure of the equipment, failure to troubleshoot and correct common problems with the equipment, or failure to remove defective equipment from service. - Environmental/Scene Factors: Factors that may result from weather conditions or factors on the ground/scene (or other). This includes temperature, light and scene safety. - Undetermined by Chart Review: The proximal cause of the adverse events (regardless of severity) cannot be determined by the information available in the chart.   The seriousness of the incident is then classified as  1. No adverse events  2. Adverse event present – potential for harm  3. Adverse event – harm identified | Sweden | Three Swedish prehospital organisations located in the Västra Götaland Region and County of Dalarna, including both urban and rural districts | Study protocol -1,080 expected | Medical record review  - Past harm | 33 |
| Hohenstein et al. (29) | Reporting system  Anonymous event reporting system | Retrospective review of events reported | Anonymous reporting system  Open Access anonymous Website from October 2005 to July 2012  Reports included information on   - Reporter and patient characteristics - NACA score - Patient conditions and problems during incident - Description of incident - Patient consequences - Reason for incident and contributing factors - Future actions / recommendations to avoid similar incidents | Germany and Austria | Emergency medicine personnel in German-speaking countries | 845 reports | Anonymous reporting system   - Harm - Anticipation and preparedness - Integration and learning | 17 |
| Holliman et al. (30) | Retrospective report review | Retrospective study | Retrospective report review – A retrospective report review was used to determine the medical command error rate in the local EMS system and to compare it with reported rates from other systems.  Cases were included only if all reviewers agreed that the case represented deviation from regional ALS protocols.  Each of the reviewed reports consisted of:   - a standard face sheet with block data sections - a narrative report - Cases of medical command error identified each month as part of quality assurance programme - Further review undertaken by study authors - Cases classified   Additional data recorded for each error case included:   - the command physician's identity and board certification status - command hospital, - paramedic identity - scene time - transport time   The run sheets were routinely completed by the paramedics  immediately after delivery of the patient to the hospital. | USA | Prehospital care in midsize city (population, approximately 78,000, in a  county with a total population of more than 300,000) in  eastern Pennsylvania.  All advanced life support run sheets reviewed in 16-month period in an urban ALS paramedic service as part of an ongoing retrospective quality assurance programme | 3,839 transports were reviewed, and errors were identified in 167 transports | Retrospective report review  - Past harm | 18 |
| Howard et al. (31) | Trigger tool  Emergency Medical Services Trigger Tool (EMSTT) | Mixed method study | EMSTT – an 8-item trigger tool was used to test the use of EMS triggers and to report on the incidence rates and types of AEs and harm they identify.  The EMS trigger tool items include:   - C1 SpO2 < 94% without supplemental oxygen or < 85% without assisted ventilation - C2 Change in systolic blood pressure >20% from first measurement - C3 Pain score > 4/10 without subsequent reduction - C4 Temperature > 38 C without subsequent reduction - C5 Increase in Early Warning Score > 1 point   Medication Triggers   - M1 Administration of opioid analgesic and Naloxone in the same patient   Procedural Triggers   - P1 Inappropriate spinal immobilization   Return-Call Triggers   - R1 Return to same patient within 24 h following refusal of transport   Records with the presence of one or more triggers were further  classified for the presence of AEs and harm.  Components of the National Coordinating Council for Medication Error Reporting and Prevention (NCC MERP) classification system were followed:   - No AE present - AE present – No evidence of harm - AE present – evidence of harm inconclusive - AE present – evidence of harm | Qatar | The Hamad Medical  Corporation Ambulance Service (HMCAS), the  government-funded national ambulance service of  Qatar and  Mecklenburg EMS Agency (Charlotte, North Carolina, USA)  All patient cases serviced by EMS division for one month meeting inclusion criteria (not reviewed as part of key clinical care pathway, no high-risk procedures, no high-risk medication, no infrequent procedures, no high-acuity interfacility transport records) | 9,836 records were reviewed | EMSTT  - Past harm | 23 |
| Howard et al. (32) | Trigger tool  Emergency Medical Services Trigger Tool (EMSTT) | Retrospective record review | EMSTT – a trigger tool was used to test the use of EMS triggers and to report on the incidence rates and types of AEs and harm they identify  The EMS trigger tool 8 items including 4 componenets:  Clinical Triggers   - C1 SpO2 < 94% without supplemental oxygen or < 85% without assisted ventilation - C2 Change in systolic blood pressure >20% from first measurement - C3 Pain score > 4/10 without subsequent reduction - C4 Temperature > 38 C without subsequent reduction - C5 Increase in Early Warning Score > 1 point   Medication Triggers   - M1 Administration of opioid analgesic and Naloxone in the same patient   Procedural Triggers   - P1 Inappropriate spinal immobilisation   Return-Call Triggers   - R1 Return to same patient within 24 h following refusal of transport   If a trigger was found, the record was further reviewed for the occurrence of AEs and/or harm. The National Coordinating Council for Medication Error Reporting and Prevention (NCC MERP) classification system was used to categorise AEs and harm  for EMSTT positive cases, and the Adverse Event Severity Rating Index developed by Patterson et al. (2012).  The Harm Classification System 1 – Modified NCC MERP   - Category A - Circumstances or events that have the capacity to cause Error - Category B - An Error that did not reach the patient - Category C/An Error that reached the patient but did not cause EMS Harm (EMS - the potential for harm to occur was present, but could not be conclusively determined based on the short duration of exposure to EMS) - Category D An Error that reached the patient and required monitoring or intervention to confirm that it resulted in no Harm to the patient - Category E Temporary Harm to the patient and required intervention - Category F Temporary Harm to the patient and required initial or prolonged Hospitalization - Category G Permanent patient Harm - Category H Intervention required to sustain life - Category I Patient death   Harm Classification System 2 – AE Severity Rating Index   - AE with Harm as a result of commission - AE with Harm as a result of omission - AE with Harm, but no fault - AE with potential to cause Harm as a result of commission - AE with potential to cause Harm as a result of omission - AE with potential to cause Harm with no fault - No AE identified | Qatar | The Hamad Medical  Corporation Ambulance Service (HMCAS), the  government-funded national ambulance service of  Qatar.  36 consecutive samples of patient care records over an 18-month period | 710 Patient Care Records sampled | EMSTT  - Past harm | 31 |
| Hoyle Jr et al. (33) | Staff survey | Cross-sectional study | Paediatric prehospital medication dosing error survey – An electronic 33-item survey was employed to assess paramedic training and practice regarding paediatric drug administration, exposure to paediatric drug dose errors and safety culture among paramedics and EMS agencies  Survey questions focused on the following areas:   - demographics and employment characteristics (age, years of EMS experience, community size, and type of EMS agency; 6 items) - paediatric training (frequency and content; 6 items) - confidence in caring for, calculating, and administering drug doses to paediatric patients (4 items) - methods used to determine paediatric patient weight (1 items) - potential solutions to make paediatric drug dosing more accurate (1 item) - knowledge of an incorrect dose of medication being delivered to a paediatric patient (1 item) - drug packaging (2 items) - the culture of safety regarding paediatric patients (8 items) - use of the Broselow Luten Tape (BLT; 4 items). | USA | Nationally certified paramedics from the National Registry of Emergency Medical Technicians’ (NREMT) database. | 1,043 completed surveys | Paediatric prehospital medication dosing error  - Anticipation and preparedness  - Reliability  - Harm | 27 |
| Ishimaru et al. (34) | Staff survey | Cross-sectional study | Occurrence of near miss events –  The occurrence of near-miss events in the last 30 days was measured as a dependent variable using a single question:  - ‘Have you experienced near misses during emergency rescues in the last 30 days? (e.g. near misses related to dropping patients, procedure/treatment errors, or careless driving)’.  Response options included:   - none - one time - two or more times | Japan | Paramedics who conducted emergency health care and disaster response at the Sapporo City Fire Department, Japan | 254 paramedics | Occurrence of near miss events  - Past harm | 24 |
| Jones et al. (35) | Standardized chart assessment tool | Retrospective chart review | Chart assessment tool - A standardized clinical chart assessment tool was created to abstract the prehospital record in order to identify the relationship between adverse events occurring during high-risk paediatric transports where red lights and sirens were used and the origin of transportation.  Adapted from hospital patient safety studies by Brennan et al and Leape et al  Safety events were classified into the following categories:   - unintended injury or consequence - near misses - suboptimal actions that are amenable to improvement - errors - management complications   The chart reviewers assigned a severity score for the potential harm associated with UNSEMs according to the following 3 categories:  - no harm likely or a near miss,  - mild or temporary harm including additional treatment, and - permanent or severe harm including death. A severe UNSEM was categorized as permanent or severe harm including death. | USA | Prehospital records of critical paediatric transports running red lights and sirens obtained from multiple EMS services spanning both fire departments and single-tier advanced life support EMS in the large metropolitan area of Multnomah County in Oregon, 2008-2011 | 490 records were reviewed | A standardized chart assessment tool  - Past harm | 26 |
| Kerner et al. (36) | Checklists  Three checklists:  -General principle of prehospital care  -Acute coronary syndrome  -Acute asthma / acutely exacerbated chronic obstructive pulmonary disease | Two-phase study, control vs implementation phases | Three checklists  To be read aloud by the paramedic and checked by the complete emergency team. Time taken always under 1 minute  General principle of prehospital care   - Past medical history - Diagnostics - Therapeutic procedures   Acute coronary syndrome   - Past medical history - Diagnostics - Therapeutic procedures   Acute asthma / acutely exacerbated chronic obstructive pulmonary disease   - Past medical history - Diagnostics - Therapeutic procedures   Subjective judgement of checklists  5 items, scale 0-100   - Checklists do not restrict my emergency medical practice - Implementation of checklists is reasonable - Checklists improve patient safety - I benefit from the introduction of checklists   Checklists lead to considerable additional effort | Germany | Missions carried out by German mobile response unit, staffed by emergency team consisting of an emergency physician (mostly board-certified anaesthetists plus a few internists) and a paramedic | 740 missions in control phase, 740 missions in implementation phase  EM Physician (n=11)  Paramedic (n=9)  ACS = total 296 Patients  Asthma/COPD = 66 patients | Checklists  -Reliability  -Reliability  -Reliability | 18 |
| MacDonald et al. (37) | Event reporting system  Decision Support Application (DSA)  Case review of mandatory reporting system and patient care records | Case review of mandatory reporting system and patient care records | DSA- An online mandatory reporting system (captures inquiries, compliments, and complaints, and report adverse events) was employed and reviewed to determine the frequency of all causes of adverse events and describe the epidemiology of adverse events.  Report completion is mandatory and occurs immediately after patient transfer.  Extraction database   - Patient demographics - Sending and receiving hospital names - Aviation details - Chronological information - special event codes - Narrative text description   The query identified any flight or patient encounter where paramedics included a report of any unusual occurrence, including patient death, aircraft mechanical problem, or weather event that took place during transport.  Reviewers independently reviewed all materials to identify adverse events. All DSA report records from Jan 01 2002 to June 30 2005  Case-finding codes used to identify adverse occurrences include:  Delay and special occurrence codes   - Ambulance delay to scene - Ambulance delay at scene - Ambulance delay to destination - Ambulance delay at destination - Delay returning to aircraft - Delay to patient contact - Vehicle delay—land en route to destination - Vehicle delay—land en route to aircraft - Delay—communication problems - Delay—due to paramedic - Delay—mechanical - Delay—weather - Delay—other - Patient expired   Special codes   - ED not ready after being notified - Pronouncement of death   Communication and patching   - Failure—obtaining patch (medical control) due to phone ⁄radio - Delay—obtaining patch (medical control) due to phone ⁄radio   Identified adverse event are then categorised using a taxonomy of event impact and event type adopted from the patient safety event taxonomy developed by the Joint Commission on Accreditation of Healthcare Organizations (JCAHO).  Taxonomy of event impact and type   - Impact: degree of harm   - No harm identified   - Possible or actual harm identified - Type: processes at fault   - Communication   - Patient management   - Clinical performance   - Medical equipment   - Transport vehicle and associated equipment   - Weather   - Unclassified | Canada | Ornge Transport Medicine, the publicly funded air medical transport system providing all air medical patient transfers in Ontario. Ornge is North America’s single largest air medical transport provider.  Air medical Transport System 26 bases carrying out approx. 17,000 patient transports annually | 1,447 web-based entries, 598 adverse events  Case finding in PCR found additional 125  Total of 723 reported adverse events  58,956 flights; 103,632 hours flown | DSA  - Past harm  Record review  - Past harm | 26 |
| McDermott et al. (38) | Retrospective record review tool | Retrospective record review | The complete prehospital and hospital records, the deposition to  the coroner, and autopsy findings were evaluated by computer analysis and peer group review with multidisciplinary discussion. The criteria used for judgments of appropriate management included those described in the Early Management of Severe Trauma Course Manual of the National Trauma Committee, Royal Australasian College of Surgeons  AIS-90 Revisions of the Abbreviated Injury Scale  Injury Severity Score  No details  TRISS methodology to estimate survival probability  No details  Errors and inadequacies were identified and categorized as  follows:   - 1. system inadequacy: failure or insufficiency of the trauma system to deliver care appropriately and timely (i.e., to provide appropriate medical/ambulance staff or facilities)   2. error in treatment/management strategy: therapeutic or diagnostic decision made contrary to available data/management plan for patient not in accordance with recommended optimal standards of practice, e.g., ATLS/EMST guidelines and those of the Victorian Ambulance Services   3. error in technique: technical error during the performance of a diagnostic or therapeutic procedure   4. error in diagnosis: injury not diagnosed because of misinterpretation, inadequacy, or lack of clinical examination or diagnostic procedure(s)   5. delay in diagnosis: diagnosis not made in a timely fashion when considered in the context of the patient's overall condition.   Deaths were classified as:   - preventable - potentially preventable - non-preventable | Australia | Records of 243 consecutive road deaths in the  Prehospital services in Victoria, Australia during 1997 and 1998 | 243 road traffic fatalities  Were reviewed | Respective record review tool  - Past harm | 20 |
| Meckler et al.(39) | Chart review tool | Retrospective record review | Chart review tool–  A chart review tool was developed to characterize the frequency, severity, and preventability of patient safety events that occur during  the out-of-hospital care of children.  Tool modelled after the Retrospective Case Record Review tool from the Harvard Medical Study, adapted to out-of-hospital setting based on results from a previous focus group study  Safety events  The instrument contained 145 questions including dichotomous answers, lists, Likert-type scales and free text.  It was divided into four sections:   1. reviewer and case information 2. medical care domains 3. summary of patient condition and UNSEMs 4. summary of case   The tool assessed:   - Unintended injury or consequences - Near misses - Suboptimal actions - Errors - Management complications (UNSEMs)   The tool was structured to identify potential UNSEMs  within specific domains of medical care:   - resuscitation - assessment, impression/diagnosis, and clinical decision-making - airway/breathing - fluids and medication - technical procedures (e.g., vascular access) - equipment; environment (scene and transport characteristics) - systems (protocols, guidelines, staffing)   Within each domain, reviewers were asked to determine whether an UNSEM occurred and the degree to which the UNSEM could have harmed the patient:   - no harm likely or a near miss - mild or temporary harm including additional treatment - permanent or severe harm including death   Degree to which UNSEM as a whole was preventable:   - 0 = impossible to prevent - 10 = entirely preventable | USA | EMS services in Multnomah County, Oregon, where both fire department and separate private transport agencies  respond to calls. | 378 charts | Chart review tool  - Past harm | 27 |
| Mortaro et al. (40) | Staff Incident Report (IR) system  Staff interview | Cross-sectional observational study | System failures were detected through an IR form (i.e., EMS failure  report form), to guide staff in reporting main system failures and to promote adequate data collection on identified critical areas.  The form included the following basic information:  1) Number identifying each medical emergency response  2) Level(s) of the process in which failure(s) occurred (phase)  3) Cause(s) of the malfunction (potential modes of error)  All data were analysed considering four different variables:   - frequency of reported interventions - phase - causes (both human and technical factors) - severity (degree of potential harm)   The form included six different phases:   - 1. emergency call   2. identification of event site   3. dispatch (severity code assignment)   4. resource allocation and timing   5. clinical evaluation and transfer   6. delayed assistance or hospital admission   Each phase included a list of potential modes of error for a  total of 30 items.  Descriptive section to report any mistakes and/or error modes not considered in the 30-item list.  Three degrees of severity were considered, following  an adapted version of the National Coordinating  Council for Medication Error Reporting and Prevention (NCC MERP) classification system for categorizing medication errors12:   - Mild: no potential consequences - Moderate: potential temporary harm - Severe: potential permanent harm or patient death   Staff interviews by the nurse responsible for data collection about their opinion of the implemented IR system.  Topics covered in interviews included:  - the effect being involved in risk management on patient safety  - error reporting  - risky behaviors or situations  - discussing mistakes within the team. | Italy | 268 day data collection period at Emergency medical dispatch centre of Verona, Italy | 142 report forms collected | IR system  - Past harm  Staff interview  -Reliaibility | 22 |
| Patterson et al. (41) | Staff survey  Emergency Medical Services Safety Attitudes Questionnaire  (EMS-SAQ) | Cross-sectional study | EMS-SAQ - 60-item survey assessing 6 domains of patient safety culture:   - safety climate - teamwork climate - perceptions of management - working conditions - stress recognition - job satisfaction   Responses to EMS-SAQ items are captured on a five-point Likert scale. (1 = disagree strongly to 5 = agree strongly).  Likert ranking is then converted to a point scale ranging from 0 to 100: disagree strongly = 0 to agree strongly = 100.  Prior efforts also dichotomized the safety domain  scores to “positive” (domain score ≥75) and “nonpositive” (domain score <75) responses. | USA | Convenience sample of staff at 61 EMS agencies  from the United States and Canada  that provided advanced life support care.  Paramedics, EMTs, first responders, prehospital nurses, EMS physicians working at least one EMS shift per week on average | 1,715 completed surveys | EMS-SAQ  - Anticipation and preparedness. | 29 |
| Patterson et al. (42) | Staff survey  Emergency Medical Services Safety Attitudes Questionnaire  (EMS-SAQ) | Cross-sectional study | EMS-SAQ - 60-item survey assessing 6 domains of patient safety culture:   - safety climate - teamwork climate - perceptions of management - working conditions - stress recognition - job satisfaction   Developed by modifying the ICU-SAQ version of the Safety Attitudes Questionnaire  Responses to EMS-SAQ items are captured on a five-point Likert scale each question (Strongly Agree to Strongly Disagree).  Likert ranking is then converted to a point scale ranging from 0 to 100: disagree strongly = 0 to agree strongly = 100.  Safety domain scores were then dichotomized to “positive” (domain score ≥75) and “nonpositive” (domain score <75) responses. | USA | 3 advanced  life support EMS agencies located in the Pittsburgh,  Pennsylvania, metropolitan area. | 71 completed surveys | EMS-SAQ  - Anticipation and preparedness. | 26 |
| Patterson et al. (43) | Framework for detecting adverse events  The Pittsburgh AE Detection and  Classification Tool (PittAETool). | Modified Delphi technique | PittAETool - A framework was developed for detecting Adverse Events associated with the treatment of critically ill or injured patients in HEMS setting.  The consensus-based framework begins with the chart reviewer (rater) applying a trigger tool (step 1), followed by the rater composing a description of the event that prompted selection of triggers (step 2). The rater then assigns a proximal cause (step 3) followed by a rating of AE severity (step 4).  Trigger tool items fell under the following categories:   - Documentation triggers (1 trigger) - Operational & Patient movement triggers (3 trigger) - Patient condition triggers (2 trigger) - Intervention and medication triggers (5 trigger)   Five categories of proximal cause were also developed (23 descriptions):   - 1. patient action—the AE was the result of action(s) by the patient;   2. actions by provider—the AE was the result of action(s) or inaction(s) by the crew (stratified into two subcategories with HEMS crew versus non-HEMS crew);   3. medical or vehicle equipment-(stratified into two subcategories with HEMS crew versus non HEMS crew);   4. environmental/scene factors—factors that may result from weather conditions or factors on the ground/scene or other, including temperature, light, and scene safety;   5. the proximal cause of the AE (regardless of severity) cannot be determined by the information available in the chart.   The severity of Adverse events was rated under three categories:   - no AE - AE present with potential for harm - AE present and harm identified | USA | Expert clinicians, senior physicians, mid-career physicians, and quality assurance officers affiliated with a large multistate HEMS organization in the Northeast US. | n/a  Tool development only, no validation presented | PittAETool  - Past harm | 24 |
| Patterson et al. (44) | Medical record review tool  The PittAETool | Experimental design comparing results between individual review and group review of medical records to detect AEs | The PittAETool  The PittAETool  Framework for performing medical record reviews  Includes trigger tool and multistep process for reviewing a medical record  The consensus-based framework begins with the chart reviewer (rater) applying a trigger tool (step 1), followed by the rater composing a description of the event that prompted selection of triggers (step 2). The rater then assigns a proximal cause (step 3) followed by a rating of AE severity (step 4).  Trigger tool items fell under the following categories:   - Documentation triggers (1 trigger) - Operational & Patient movement triggers (3 trigger) - Patient condition triggers (2 trigger) - Intervention and medication triggers (5 trigger)   Five categories of proximal cause were also developed (23 descriptions):   - 1. patient action—the AE was the result of action(s) by the patient;   2. actions by provider—the AE was the result of action(s) or inaction(s) by the crew (stratified into two subcategories with HEMS crew versus non-HEMS crew);   3. medical or vehicle equipment-(stratified into two subcategories with HEMS crew versus non HEMS crew);   4. environmental/scene factors—factors that may result from weather conditions or factors on the ground/scene or other, including temperature, light, and scene safety;   5. the proximal cause of the AE (regardless of severity) cannot be determined by the information available in the chart.   The severity of Adverse events was rated under three categories:   - no AE - AE present with potential for harm   AE present and harm identified | USA | Emergency clinicians; included prehospital flight nurses, flight paramedics, and emergency medicine physicians.  Two random samples of medical records from 2008 from a large HEMS agency in the USA. First sample of 50 included equal numbers from each month of 2008. Second sample of 20 included 10 records with a pre-existing quality assurance marker. | 70 records reviewed  50 randomly selected chats reviewed by RN/Medics (n= 3)  Mid-career emergency physicians (n=3)  20 records discussed to reach consensus based AE decisions | The PittAETool   - Harm | 30 |
| Patterson et al. (45) | Patient Care Report review | Tool / consensus development (Delphi) plus chart review study | Chart review –  Raters applied the following definition when reviewing Patient Care Reports (PCR) to identify Adverse Events in Ground Transport Emergency Medical Services.  Developed by iterative consensus (Delphi-like) process  The consensus definition was as follows: “An adverse event in EMS is a harmful or potentially harmful event occurring during the continuum of EMS care that is potentially preventable and thus independent of the progression of the patient’s condition.”  Then the severity of identified adverse events was rated using the Patient Adverse Event Severity Rating Index:  1) AE with harm as a result of commission  2) AE with harm as a result of omission  3) AE with harm, but no fault  4) AE with potential to cause harm as a result of commission  5) AE with potential to cause harm as a result of omission  6) AE with potential to cause harm with no fault  7) No AE identified | USA | 3 ground transport EMS agencies located  in the midwest and northeast US Census regions. | 250 charts were reviewed | PCR review and AE severity Index  - Past harm | 31 |
| Patterson et al. (46) | Staff survey  The EMS Safety Inventory (EMS-SI). | Cross-sectional study | EMS-SI - 44-item survey that measures self-reported safety outcomes from EMS workers.  Developed through Delphi-style consensus process, items are classified into one of three safety domains using the Agency for Healthcare Research and Quality (AHRQ) Patient Safety Indicator tool:   - provider injury (2 items) - medical errors or AEs (25 items) - safety-compromising behaviours (17 items)   Item responses scored on two scales:   - 7-point Likert scale (definitely not to definitely yes, plus n/a and don’t wish to answer) - “ran out of time,” “forgot to perform,” “not part of protocol,” “did not think it necessary,” “contraindicated,” “do not wish to answer,” and “not applicable to me.” | USA | EMS managers affiliated with an EMS management group with a total membership of 2,253 managers (the National EMS Management Association) four U.S.  Census regions. | 511 completed surveys | EMS-SI  - Past harm (provider injury, medical errors or AEs, & compromising behaviours subscales)  - Reliability (safety compromising behaviours subscale) | 28 |
| Price et al. (47) | Staff survey | Cross-sectional study | A 12-item survey was developed to assess and understand patterns in adverse events in prehospital care.  Paramedics were asked to   - Consider a problematic case encountered where something went wrong, something was going to go wrong but was salvaged, or a mistake was made without adverse consequences - AEs of all types and severity sought - Indicate which components they felt contributed to the case   OAdditional questions were also ased:   - Paramedic cognition - Incident reporting - Recovery from incidents - Severity of outcome - Paramedics’ clinical practice level   71 contributing factors across 5 domains:   - Patient - Scene - Organisation - Workload - Paramedic | Australia | Frontline staff of the Ambulance  Service of New South Wales (ASNSW), a large  Australian ambulance service which responds to  approximately 1.1 million emergency calls per year,  across a vast geographical expanse (800 000 square  kilometres), encompassing both metropolitan and  remote areas. | 370 surveys completed | Survey of AEs  - Past harm | 23 |
| Rasmussen et al. (48) | Staff survey | Cross-sectional study | An 8-item survey was used to assess perceived patient and flight safety in HEMS systems during various mission types  Two domains were assessed:   - patient safety (1 items) - flight safety (3 items)   Respondents evaluated patient and flight safety  during various mission types in their own service on a 7-point symmetric Likert scale, ranging from “totally unacceptable” (1) to “perfectly acceptable” (7). | Norway | Medical directors of HEMS in Europe, North America, Australia, New Zealand, and Japan. | 66 surveys were completed  113 responses  66 eligible for analysis  17 Scandinavia  28 Rest of Europe  17 North America  3 Australia  1 Japan | Survey  - Anticipation and Preparedness | 25 |
| Ray et al. (49) | Medical Examiner record review criteria | Retrospective record review | A panel of two board-certified attending Trauma and Critical Care Surgeons reviewed medical examiner records to determine if injuries were potentially survivable.   1. Potentially Preventable Death (PPD) was assigned when death may have been avoided had optimal medical therapy been immediately available based on previously outlined criteria.   These criteria include:   - severe anatomic injuries potentially survivable under perfect circumstances and perfect resuscitation and - absence of anatomical non-survivable injuries.   PPD included, for example, non-devastating traumatic brain injury such as epidural or subdural haemorrhage, and haemorrhage from solid organ injuries that were deemed surgically treatable.   1. Non-Preventable Death (NPD) included cases with devastating   injuries with anatomical destruction not survivable even under perfect circumstances and resuscitation.  These included injuries such as:   - major rupture of the heart - uncontained laceration or transection of the aorta or thoracic vena cava - massive intra-parenchymal brain tissue trauma - atlanto-occipital dislocation or brainstem herniation - severe charring due to massive burns. | USA | Medical Examiner records for 2011 for all pre-hospital deaths of occupants of 4-wheel motor vehicles involved in collisions, excluding pedestrian and motorcyclist deaths in Miami-Dade. | 98 deaths for occupants  of 4-wheeled motor vehicle collisions were reviewed | Record review  - Past harm | 16 |
| Rosenfield et al. (50) | Retrospective record review | Retrospective record review | Retrospective record review – A review of all Victorian road traffic deaths was conducted to the medical management of patients who died following motor vehicle accidents.  Clinical findings and management were recorded according to a comprehensive computerised data proforma detailing:   - The patient - The accident/mechanism of injury - Pre-hospital: assessment, management and transport - Emergency room (ER): reception, resuscitation, investigation and specialist review - Operating room: surgical and anaesthetic procedures - Intensive care unit (ICU) management - Ward/high dependency unit (HDU) management - Inter-hospital transfer - Complications - Clinical diagnosis of injury - Causes of death - Autopsy diagnosis of injury and causes of death.   Injury type and severity were coded using both AIS-85 and AIS-90 Revisions of the Abbreviated Injury Scale. AIS 85 codes  were used to derive the Injury Severity Score (ISS). Audit filters  based on those of the American College of Surgeons Committee  on Trauma were applied. A narrative account of the patient’s  management was prepared. Survival probability was estimated by  TRISS methodology using AIS 85 codes.  An evaluative committee examined each area of care of the patient’s management and individual problems and their contribution to death identified.  Problems were identified and categorised as follows:   - System inadequacy: failure or insufficiency of the trauma system to deliver appropriate and timely care as a result of inadequate facilities or personnel - Management/treatment error: a therapeutic or diagnostic decision made contrary to available data, or a management plan for the patient not in accordance with recommended optimal standards of practice - Technique error: a technical error occurring during the performance of a diagnostic or therapeutic procedure. - Error in diagnosis: diagnosis missed because of misinterpretation, inadequacy or lack of physician examination or diagnostic procedure(s) - Delay in diagnosis: a diagnosis not made in a timely fashion when considered in the context of the patient’s overall condition.   The criteria for judgement of appropriate management were based  on the Early Management of Severe Trauma (EMST) Course  Manual of the National Trauma Committee, Royal Australasian  College of Surgeons.  Deaths were classified as  - potentially preventable (pp),  - preventable (p), or  - non-preventable. | Australia | Records of 449 fatalities due to road traffic deaths in Victoria, Australia | 449 fatalities assessed | Record review  - Past harm | 17 |
| Salerno et al. (51) | Quality assurance tool  Incident report system | Retrospective consecutive case series.  Prospective incident reporting study | A quality assurance tool was used determine the incidence, type, and outcome of protocol deviations in an emergency medical services (EMS) system.  ALS runs over a 2 month period were reviewed for protocol deviations  Prehospital care reports were reviewed to obtain the following information:   - the nature of the problem - the ambulance service - the time spent on scene - the completeness of the history - the protocol violations involved   If a deviation in protocol was found, a review was conducted, and the deviation was assigned to one of three categories;   - minor (no potential for significant adverse effects), - serious (potential for significant adverse effects), or - very serious (likely to cause significant adverse effects).   When protocol deviations occurred, the course of each patient involved was reviewed using ED and inpatient records.  A deviation was graded "positive" if an improvement was documented, "negative" if the patient deteriorated, and "no effect" if changes in patient status were not documented as a result of the deviation. | USA | Seven advanced life support ambulance services servicing five  area hospital emergency departments servicing Monroe and  Livingston counties in New York | 1,246 runs were reviewed, 199 contained deviations  1246 Patient requiring ALS; 16% had deviations   - 55% minor - 38% serious - 7% very serious | Quality assurance tool  - Past harm  - Reliability  Second measure? | 17 |
| Shaw et al. (52) | Incident reporting system | Retrospective review | Incident reporting system -  The data for each incident were checked for the following data sets:   - time of incident - date of incident - patient age - patient sex - clinical specialty - location - risk rating - outcome for patient - type of incident - description of incident   The outcome was then graded under the following categories:  - catastrophic  - major  - moderate  - minor  - none | UK (England and Wales) | Staff from 18 NHS trusts in England and Wales (12 acute trusts, three mental health trusts, two ambulance trusts, and one primary  care trust) | 124 ambulance reports (out of 28,998) | Incident reporting system  - Past harm | 22 |
| Sørskår et al. (53) | Staff survey  Norwegian Prehospital Survey of Patient Safety Culture (PreHSOPSC) | Cross-sectional study | The PreHSOPSC -  A 46-item instrument was used to assess culture of patient safety in prehospital settings under the following 13 dimensions:  Plus two single-item outcome items   - Patient safety grade - Number of events reported in last 12 months   Dimensions:   - Outcome dimensions   - Overall perception of safety (4 items)   - Frequency of error reporting (3 items)   - Stop working in dangerous situations (4 items) - Safety climate dimensions – unit level   - Manager expectations and actions promotion patient safety (4 items)   - Organisational learning – continuous improvement (3 items)   - Teamwork within units (4 items)   - Communication openness (3 items)   - Feedback and communication about errors (3 items)   - Nonpunitive response to error (3 items)   - Staffing (4 items) - Safety climate dimensions – system level   - Hospital management support for patient safety (3 items)   - Teamwork across units (4 items)   - Handoffs and transitions (4 items)   The response format ranges from 1 (disagree strongly) to 5 (agree strongly) on a Likert scale. | Norway | Prehospital personnel in the Norwegian GEMS and HEMS, recruited through contact with prehospital system leaders | 1387 surveys completed | PreHSOPSC  - Anticipation and Preparedness | 35 |
| Sorskar et al. (54) | Staff survey  The Prehospital Survey on Patient Safety Culture short version (PreHSOPSC-S) | Tool validation study | The Prehospital Survey on Patient Safety Culture short version  (PreHSOPSC-S)  Developed based on an adjusted short version of the Hospital Survey on Patient Safety Culture instrument  Six dimensions, 17 items   - Management support for patient safety (2 items) - Manager expectations and actions promoting patient safety (2 items) - Teamwork within units (3 items) - Learning, feedback and improvement within units (3 items) - Stop working in dangerous situations (3 items) - Transitions and handoffs (4 items)   Items scored on 5-point Likert scale | Norway | HEMS and ground ambulance personnel | 2012: 172 Responses; 145 completed  2016: 118 Responses; 108 completed  GEMS  2016: 1269 Responses; 1045 completed  Questionnaire completed in 17 out of the 18 Health Trusts | The Prehospital Survey on Patient Safety Culture short version  (PreHSOPSC-S)  -Anticipation and preparedness | 36 |
| Stella et al. (55) | Event monitoring system  Critical Incident Monitoring System  Chart review | Prospective, descriptive study  Prospective incident monitoring study | Incident monitoring combined with chart review was employed to describe the nature of critical incidents in prehospital care. Anonymous reporting was available to ambulance staff via a Web-based reporting system in addition to a paper-based version.  All major trauma cases as defined by Department of Human Services (DHS) criteria  Used predefined template to collate relevant facts from chart review when issues arose  “Hot debrief” protocol - Offered to participants involved in selected major trauma cases  Interviews with ambulance staff to elucidate detail or undocumented incidents  Conducted by senior ambulance representative and consultant emergency physician  Conducted within 14 days of event  All cases were examined for incidents, which were further classified and categorized using a structured format. Incidents initially were classified into 11 Critical Incident Monitoring System (CIMS) Incident types:   - Communications - Prolonged times - Resources - Equipment - Resuscitation - Other treatment problem - Deviation from CPG - Diagnostic error - Interference - Injury - Other   They were further classified based on the system employed by the CCRTF study into 6 CCRTF categories:   - System - Inadequacy - Management - problem - Technique - Diagnosis delay - Diagnosis error - Unknown   Incidents also were assessed for the risk from adverse outcome using a frequency and severity of outcome matrix.  These included the following 6 domains:   - None/Near miss - Minor - Moderate - Major - Death - Unknown   Consultative Committee on Road Traffic Fatalities engaged and classified identified related to system and management e.g. “Prolonged time at scene” for Management  Counted by numbers mitigated and not mitigated | Australia | Rural Ambulance Victoria ambulance Area 1 (Barwon South West Region) in South Western Victoria, serving a population of >240,000. | 230 cases reviewed, 454 incidents identified | CIMS  - Past harm  Chart review  -Past harm | 26 |
| Stella et al. (56) | Event monitoring system  Critical Incident Monitoring System  Chart review | Prospective, descriptive study  Prospective incident monitoring study | Incident monitoring combined with chart review was employed to describe the nature of critical incidents in prehospital care. Anonymous reporting was available to ambulance staff via a Web-based reporting system in addition to a paper-based version. An initial chart review of the ambulance patient care records and hospital notes was conducted.  “Hot debrief” protocol  Offered to participants involved in selected major trauma cases  Interviews with ambulance staff to elucidate detail or undocumented incidents  Conducted by senior ambulance representative and consultant emergency physician  Conducted within 14 days of event  Each case was examined for the presence of critical incidents, and each incident was categorized using a structured  format. The incident initially was categorized to one of 11  "CIMS Incident Types":   - Resource problems - Communication problems - Prolonged times - Resuscitation problems - Other treatment problem - Equipment problems - Other - Injury - Deviation from CPG - Diagnostic error - Interference   The incident was classified further based on the system employed by the CCRTF study into 6 categories:   - System - Inadequacy - Management - Technique - Diagnosis delay - Diagnosis error   Each incident was assessed for risk of adverse outcome using a matrix that related incident frequency to severity of outcome.  These included the following 6 domains:   - None/Near miss - Minor - Moderate - Major - Death - Unknown   Consultative Committee on Road Traffic Fatalities engaged and classified identified related to system and management e.g. “Prolonged time at scene” for Management  Counted by numbers mitigated and not mitigated | Australia | 104 personnel involved in the Rural Ambulance Victoria ambulance Area 1 (Barwon South West Region) in South Western Victoria, serving a population of >240,000. | 41 cases reviewed | CIMS  - Past harm  Chart review  - Past harm | 22 |
| Wang et al. (57) | Retrospective insurance claim review tool | Retrospective review of claims | Retrospective review of insurance liability claims –  Claim data was collected from the insurance company’s  computer and paper records.  Status   - Open - Closed - Unknown   Legal outcome   - payment to claimant - out-of-court settlement - trial verdict   A computer database contained key aspects of each incident including   - claimant and insured identities, - basic policy information, - financial information (including actual or estimated total incurred costs), and - a brief description of the event.   Potential cases were identified by first searching the case descriptions of the computer database.  The abstractors performed a structured chart review using a standard, confidential, laptop computer– based data collection instrument, with drop-down boxes to help standardize responses.  The following information was identified from the review:   - the status (open, closed, or unknown) and legal outcome (payment to claimant, out-of-court settlement or trial verdict) of each claim, - general characteristics of each incident, including the age and sex of the claimants, the type of emergency vehicle involved (ground ambulance, wheelchair or stretcher van, fire department vehicle, air medical helicopters EMS first response vehicle), and - mode of emergency response (emergency versus nonemergency).   The general category of each adverse event was identified and classified as:   1. emergency vehicle crash or movement (included vehicular crashes and incidents resulting from movement of the vehicle) 2. patient handling (instances in which the stretcher or wheelchair “tipped,” in which the patient fell or was dropped by emergency personnel, and events in which the patient was otherwise injured during movement or handling) 3. clinical management (adverse drug events, airway management events, procedural or medical decision incidents, or the absence or malfunction of key medical or rescue equipment) 4. response or transport (EMS dispatch, response, navigation to or from the scene, or delay of care) 5. other events   The injuries reported for each event were identified,  categorizing these outcomes as   - death, - life-threatening, or disabling injuries and - non–life-threatening/other/unknown injuries (emotional, death or injury to a fetus, and other). | USA | EMS liability  insurance claims data from McNeil and Company, Inc,  Cortland, NY including coverage to both private  and public emergency response agencies nationally, including  ambulance services, fire departments, and medical transport  companies, among other operations  Sample of EMS liability insurance claims data from one insurer in New York state. All tort claims filed during 24-month period | 326 claims were reviewed | Retrospective review  - Past harm | 29 |
| Weaver et al. (58) | Staff survey  The EMS Safety Attitudes Questionnaire (EMS-SAQ).  Staff survey  The EMS Safety Inventory (EMS-SI). | Cross-sectional study | EMS-SAQ - 60-item survey assessing 6 domains of patient safety culture:   - safety climate - teamwork climate - perceptions of management - working conditions - stress recognition - job satisfaction   Responses to EMS-SAQ items are captured on a five-point Likert scale.  EMS-SI - 44-item survey that measures self-reported safety outcomes from EMS workers. The EMS-SI was developed by a panel of EMS medical directors, emergency medical technicians (EMTs) and paramedics, and occupational epidemiologists to identify events or behaviours with harm or risk of harm.  Items are classified into one of three safety domains using the Agency for Healthcare Research and Quality (AHRQ) Patient Safety Indicator tool:   - provider injury (2 items) - patient care error or AE (25 items) - safety-compromising behaviours (17 items)   Nominal, seven-option, categorical scales were used to elicit responses to EMS-SI items. | USA | EMS leaders and workers in America affiliated with a convenience sample of agencies recruited from a national EMS management organization | 416 | EMS-SAQ  - Anticipation and preparedness.  EMS-SI  - Past harm  - Reliability | 24 |
| Yardley et al. (59) | Staff incident reporting system  The National Reporting  and Learning System (NPLS) | Retrospective incident review | NPLS –  Each structured reporting form collects information on   - demographic and administrative data - the circumstances of occurrence - a categorisation of causation - an assessment of the degree of harm as ‘none’, ‘low’, ‘moderate’, ‘severe’ or ‘death’ - action taken or planned to investigate or prevent a recurrence   These data are captured in a structured reporting form with a free-text field where the reporter is asked to describe what happened and why they think it happened.  Each reviewer categorised each incident report according to the main event that had caused the harm and also identified a larger number of primary failures that had contributed to the main event.  Each incident report was coded into a single-event category with at  least one primary failure.  Three event categories emerged from the coding:   - 1. patient injury: incidents where a patient died following physical injury while in the care of the ambulance service   2. delay in response: incidents where the time taken to reach a patient was excessive   3. failure to provide necessary care: incidents involving shortfalls in standards of care after ambulance service personnel had contact with the patient   Underlying these three types of event were 12 primary failures, 9 of which were organised into two groups of related causes  ▸ Equipment  – Faulty equipment  – Misused equipment  – Missing equipment  ▸ Error in dispatch  – Unable to receive 999 call  – Unable to locate call  – Miscommunication  – Automated dispatch system failure  – Poor prioritisation  – Vehicle/crew shortage  ▸ Weather  ▸ Patient characteristics  ▸ Clinical misjudgement | UK (England and Wales) | Staff at NHS organisations in England and Wales | 69 incident reports from ambulance services | NRLS  - Past harm | 27 |
